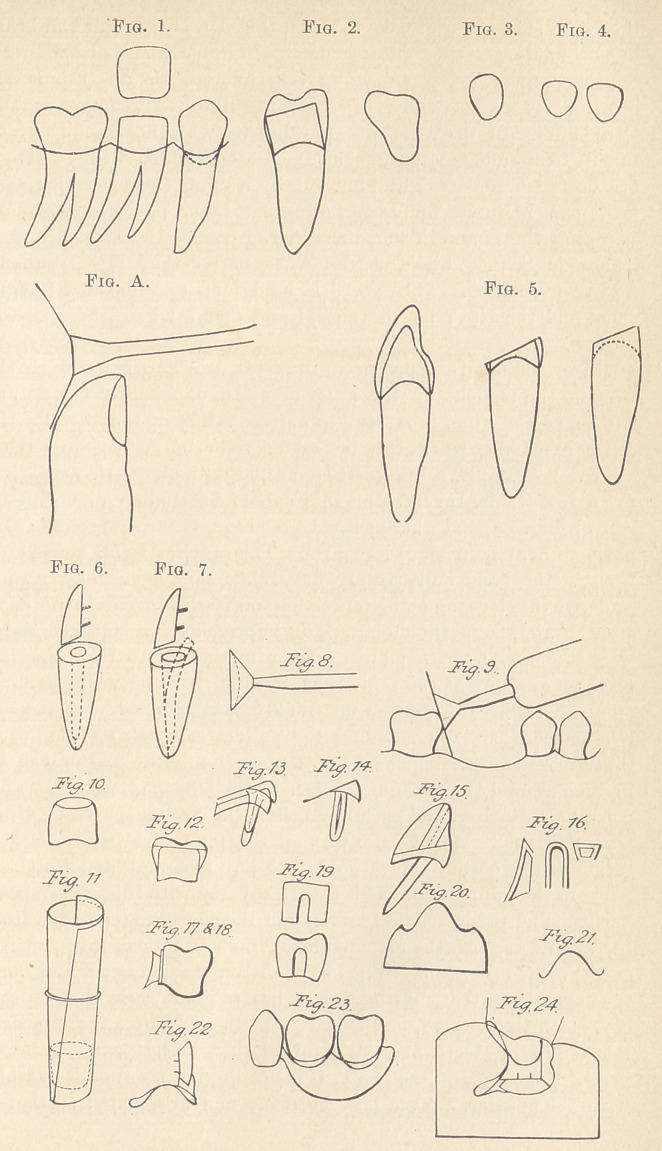# Crown- and Bridge-Work

**Published:** 1897-05

**Authors:** Fred. A. Peeso


### International Dental Journal.

Vol. XVIII. May, 1897. No. 5.

### Original Communications.[1]

### CROWN- AND BRIDGE-WORK.[2]

BY FRED. A. PEESO, D.D.S.

Mr. President and Gentlemen,—I feel greatly honored in having been asked to read a paper before this society. With full appreciation of the courtesy extended to me, it was nevertheless with considerable trepidation that I undertook to comply. While I may not have much to present to you which is strikingly new and original, I have written with the hope that some new light might be thrown on old ideas.

It would be presumption on my part to come before you in the attitude of a teacher. I prefer to be looked upon as a fellow-student in considering the subject which has been assigned me, and if in the reading of this paper and the discussion which follows you may be able to glean some points of value from me, and I from you, I shall esteem myself amply rewarded. The methods which I shall attempt to describe have proved most satisfactory in my practice, some of them having cost me much hard work both at the chair and in the laboratory. Crown- and bridge-work is one of the most important branches taught in our schools, for

[1] The editor and publishers are not responsible for the views of authors of papers published in this department, nor for any claim to novelty, or otherwise, that may be made by them. No papers will be received for this department that have appeared in any other journal published in the country.

[2] Read before the New York Institute of Stomatology, February, 1897.

the reason that more harm may result to patients from its improper use than from any other cause.

While comparatively new, it has still been long enough before the public to prove that it has its place in dentistry. It has perhaps caused more controversy than anything connected with the profession,—and the arguments for and against are both right and wrong. While some enthusiasts claim everything for it, others go so far as to say that it has no place at all in dentistry, and maintain that it is malpractice to use it.

Few new ideas have been presented that have not at first been scoffed at, but most of them have proved in some way useful; and often this very antagonism promotes their growth and brings about the desired result. This has been the case with bridge-work. The opposition it has had to contend with from the start has caused those interested in it to strive to eliminate the weak points, until to-day it is a most valuable adjunct to the profession. To make the work successful, good judgment and skill are required on the part of the one doing it. The principal elements entering into a successful operation are, first, a healthy condition of the mouth and teeth or roots to be used as abutments; second, their proper preparation to receive the attachments; third, accurate fitting of caps and bands; and, fourth, perfect articulation. Any one of these conditions ignored, failure may ensue. The thought of wearing a plate is repugnant to most people, but the same prejudice does not exist against bridge-work. If instead of a partial plate one or more bridges be placed, there is nothing that will please the patient more or give him more lasting satisfaction. A fine filling or a plate may gratify, but not as a nicely finished and well-fitted bridge. Unquestionably a large part of the work done is not only unfit to be put in the mouth but is a positive injury to the patient.

Before advising the placing of a bridge care should be taken to see that the teeth are in good condition and sufficiently strong to do the work imposed. Too much strain should not be put on a single tooth or root. Ordinarily two good roots will support a bridge of three or four teeth, and I have seen satisfactory cases of even five that have been in for a number of years, but that is rather more than is generally advisable. I am speaking now more particularly of permanent fixtures. It is perfectly safe to put a full denture on the two cuspids and the two first or second molars. A piece of this kind is very strong, as there is little lateral motion, and each side helps to support the other. A cuspid and first molar and very often the second molar will form safe anchorages. The two centrals will carry the two laterals and the laterals the centrals. Frequently the canines may serve to carry the four incisors if they stand well apart, and the teeth may be placed nearly in a straight line, but if the arch is narrow and very much curved the leverage would be so great as to eventually loosen the abutments. Removable work gives much wider field of operation, as by the employment of the saddle a piece may be extended beyond the anchorages. This is especially true of the lower, where by utilizing the bicuspids or cuspids most of the posterior teeth may be restored, particularly if there is a good ridge.

In the preparation of the teeth and roots the greatest care should be used; for if not properly done the chances are against the work proving satisfactory. While the trimming of the teeth seems like a simple operation, it is by no means easy. The operator should always have in mind what the shape of the tooth would be if it were cut across just below the gum line. The swell should be entirely taken off to about one-sixteenth of an inch below, leaving the sides parallel, or slightly larger at that point, so that when the band is passed over it will hug the neck tightly. If the tooth is larger near the cusp than below the gum, the band, when put on, instead of passing between the tooth and gum cuts into the gum, and when cemented the cement will present a rough, jagged surface which will be a constant source of irritation. An explorer should be used constantly, and trimming should continue as long as the least particle of enamel or the least ridge can be felt. In all of the teeth the bulk of the trimming will be on the mesial and distal surfaces, the swell being greater at those points.

Looking down on a lower molar after it has been shaped, it will be seen to be nearly square, with the corners rounded, being slightly wider at the mesial than at the distal side, owing to the anterior root being larger. (Fig. 1.) The upper molars will be somewhat triangular, being broader on the buccal side because of the two buccal roots being larger than the palatal. Occasionally this may vary, but not often. (Fig. 2.) In the bicuspids, cuspids, and laterals the roots are egg-shaped, with the base towards the labial side (Fig. 3), the bicuspid being long and narrow.

The shape of the centrals is always nearly a perfect triangle with rounded corners. (Fig. 4.)

In any of the anterior teeth, if the enamel be entirely removed, the root will be of the proper shape to receive the band, as the greatest circumference of the body of the tooth is at the junction of the enamel with the dentine. (Fig. 5.)

In opening a canal in such a tooth, it will be found that if its direction be not changed the pin will come wholly or partly under the facing, which necessitates the grinding away of the pin so as to leave it attached only to the thin floor of the cap, or grinding the facing. (Fig. 6.) If in enlarging the canal the reamer be pressed towards the palatal side of the root, thus sloping the canal in that direction, and then, by bending the pin slightly, plenty of room will be left in front of it for the facing. (Fig. 7.) The pin, too, should be long and heavy enough to support the crown. Very frequently a patient presents to have a crown reset, perhaps a large cuspid or central, having a pin not more than three-sixteenths or one-fourth of an inch in length and of No. 16 or 17 wire, where No. 13 or 14 of three-eighths or one-half inch in length could have been used. In opening the canal, the length should first be ascertained by passing a fine broach through the apical foramen, the pin then being made long enough and large enough to give all the strength required. In looking after details like these much time may be saved and success assured.

The instruments used for the preparation of the teeth and roots are numerous, and all have their advocates. Those I have found most useful are the following:

For grinding down the cusps for metal crowns, or any of the anterior teeth for porcelain-faced crowns, a square-edged corundum or carborundum wheel is probably the best. For trimming the sides of molars and bicuspids a diamond disk or thin corundum disk is indispensable, and for rounding corners and parts that cannot be reached with the disks, a little cup-shaped corundum (S. S. White's No. 11) is necessary. (Fig. 8.) I had some steel cutters made after the No. 11 model, cut same as finishing burs, which do the work well. Perhaps the most difficult place in the mouth to reach is the anterior surface of the lower molars. For this it is necessary to have a saucer-shaped disk, as with the ordinary straight wheel the angle is such that the tooth cannot be trimmed at the neck without cutting far back into the crown. (Fig. 9.) By taking a thin mounted corundum, holding it near the flame of a spirit lamp or Bunsen burner, and pressing the thumb or a stick against the back while revolving rapidly, the wheel will be softened and a concave disk formed which will answer every purpose. (Fig. A.)

After a tooth for a porcelain-faced crown has been ground down, for levelling the surface and cutting it below the gum margin at

FIG. 1.

FIG. 2.

FIG. 3.

FIG. 4.

FIG. A.

FIG. 5.

FIG. 6.

FIG. 7.

Fig. 8.

Fig. 9.

Fig. 10.

Fig. 13.

Fig. 14.

Fig. 15.

Fig. 12.

Fig. 16.

Fig. 11

Fig. 19

Fig. 20.

Fig. 17 & 18.

Fig. 21.

Fig. 23.

Fig. 24.

Fig. 22

the labial side, the root-facers, designed by Dr. Ottolengui and bearing his name, are the only instruments suitable, and will hardly be improved upon. For enlarging the canals I use an instrument bearing my name, which is something like the reamers of Dr. Ottolengui, but the sides are parallel to near the end, where it tapers to a safe point, same as the Gates-Glidden drills. They are designed for straight pins, but by leaning them to either side they can be used for Logan or other taper pin crowns. For stripping the enamel from the anterior roots, the Nos. 3 and 7 scalers of S. S. White's do the work better than any trimmers I have ever used. The No. 3 is used for the labial and palatal sides, and the No. 7 on the mesial and distal, where the space is narrow.

After the mouth is prepared, the work can be wholly or partly done on the model. In the anterior teeth, or where the roots are cut below the gum, it is best to fit the bands in the mouth. In other cases the work can be better done on a plaster model. Take, for example, a bridge from cuspid to molar. In preparing for a removable, the attachments should be slightly diverging or converging, so that there may be a slight natural spring of the parts to help hold the piece firm. This applies to all removable work. The band and cap are first made for the cuspid, the tube soldered in place, and an impression taken, being careful to have an accurate one of the molar. The impression can be taken and a wax bite used; but a more accurate method is to take the impression and bite at the same time, by first covering well the abutments, building the plaster up thickly, taking in a few of the adjoining teeth, and then having the patient close the mouth, biting into the soft plaster. Then with a wet spatula the plaster can be pressed around the teeth and gums. After the impression has become hard remove, and carefully fasten the broken parts together with hard wax. Put a little wax on the pin and around the sides inside of the cuspid cap to obliterate any undercut, and fasten in place in the impression. This is then covered with colored sandarac varnish, and the lower side run, letting the plaster extend back from the molars to form the articulator. Notch or groove this, varnish, and run the other side. When hard the impression is cut away and the parts separated. With a pair of heated pliers remove the cuspid cap, clean the wax off, and replace it. In this way a more perfect relation of the teeth may be obtained than with a wax bite, as in pressing the wax on the model it is apt to give more or less, while with the plaster, a piece may be made and articulated so that it will not have to be touched when placed in the mouth, and if necessary a second bridge could be made from it.

The model should be thoroughly dried with slow heat, and the molar trimmed evenly all around to nearly one-sixteenth of an inch below the gum line, being careful to cut parallel to the sides of the tooth. Then give it several coats of very thin sandarac varnish, which will soak in readily, and when it has taken all that it will, dry thoroughly, and the model will become so hard that the fitting of the band will not mar it.

There are various kinds of attachments used in removable work, the most common of which and the most easily made is the telescope cap, which we will first consider. Its name indicates the principle on which it works, and a few words will explain how it is made. The model being ready, the measurement of the tooth at the gum line should be taken with wire, or an annealed copper strip, which is preferable. Cut the gold a little longer to allow for the lap and on a slight angle so as to make the band a little larger at the neck. Bevel one or both ends, and sweat or solder together with twenty-one-carat solder. After festooning it to follow the gum line, cut off to a point nearly level with the top of the tooth. Press the edges inward all around (Fig. 10), file off even with the tooth, and solder on the floor, using plenty of solder. The corners may then be rounded and the cap polished. Now to make the outer movable cap. First put a thin film of wax on the inside of the cap already made, roll a piece of paper around a pencil or instrument of suitable size, and push the cap through it to near the end. Put a small rubber band around to hold it together and fill with fusible metal, of which mention will be made later. (Fig. 11.) This makes the cap solid, and it can be worked on without injury. Measure with copper strip, cut the gold so that the band will be a little small, bevel the ends to form a smooth joint inside, and sweat or solder with twenty-one-carat solder. The joint may be smoothed on the beck-horn of an anvil and driven on to the supported cap. This gives a perfect fitting telescopic cap such as can be got in no other way. The top should then be pressed in to conform to the inner cap, filled even with it, and a floor soldered to it with twenty-one-carat solder. The lower edge should be parallel with the lower edge of the inner cap, but about one-sixteenth of an inch above it, so that it will come only to or not quite to the gum line. A contour may then be given by soldering pieces of gold to the sides (Fig. 12) and filing off even with the top. A cusp is then selected and soldered on with twenty-carat solder. When the whole is finished it appears to be an ordinary contoured, full-gold crown. (Fig. 12.) The inner cap is then warmed slightly and the fusible metal pulled away. Any particle of it adhering to the cap should be carefully scraped out and the cap dipped in nitric acid to dissolve any of the metal remaining.

A solid cusp is necessary in this case, and is, I think, preferable in all cases. It makes a stronger crown, will wear better, and be the same color as the band. It is easily made by swaging a very thin piece of pure gold for a matrix and filling with coin or scraps from bands. It may also be made by casting.

For a high-grade solder it has been my custom to melt together a pennyweight of coin gold and six grains of S. S. White's eighteen-carat solder, which makes nearly twenty-one carat and of a color hardly to be distinguished from coin. It requires care in manipulating, but after a little practice it can be worked nicely.

For the cuspid, two or more kinds of attachments may be used. In using a split pin and tube, the cap is made as for a Richmond crown; but in place of a pin, a tube is used which may be made by drilling a No. 14 hole in No. 12 platinum and iridium wire on a jeweller's lathe, or by bevelling the edge of a piece of platinum and iridium plate and rolling it around a piece of wire of the right size, soldering with pure gold without flux to prevent gold from flowing into the tube, driving through it a steel wire slightly larger than the one it was rolled on and closing the end and soldering with pure gold. After the cap has been made drill a hole into the floor over the enlarged canal, put the tube in, fasten with hard wax, remove, invest, and solder, using plenty of solder in order to strengthen attachment to under surface of floor, and allow for countersinking from upper surface. Then face off on a smooth corundum wheel or very fine file, and slightly countersink the opening into the tube. (Fig. 13.) To make the removable part, fit a partial band to the palatal side of the cap extending only to or not quite to the gum line, and on the sides to about where the facing will come. (Fig. 13.) Solder to it a floor, covering the whole cap with twenty-one-carat solder.

Drill through this into the tube, wax the split pin in position, invest and solder with twenty-carat solder. The caps are then placed in position, and if no saddle is to be used, the bridge may be made the same as a stationary piece, waxing to the outer caps. The split pin is made by bending together a piece of half-round platinized gold wire, and turning or filing to fit the tube. It is well to leave the end closed, and it may be spread a little in the middle with a fine sharp instrument, thus forming a very close elliptic spring, and there is no danger of one side being split away. (Fig. 14.)

Another form of attachment is to let the pin extend through the floor of the cap, bend it so as to bring it nearly in line with the other attachments, fit a tube to it, and solder it in the back of the canine. (Fig. 15.)

A key may also be used, but it is hardly as suitable in connection with a canine as the methods already described. For molars and bicuspids it is excellent and is easily made. Take a piece of platinum-iridium, file it into the required shape, which is something like this (Fig. 16). Bend a piece of the same metal, about thirty-two gauge, around the edges, fitting the sides perfectly. (Fig. 16.) File off even with the broad side of the key, fit a floor to it, and solder with a very little pure gold without flux. The male and female parts are now ready. In using this, the side of the crown to which it is to be attached should preferably be straight and of double thickness. (Fig. 17.) The key is put in position, and a hole drilled through and fastened with a small rivet, having first flowed over the side to be attached to the cap a thin film of pure gold, as the union between this metal and solder is not strong. It is then soldered in position. (Fig. 18.) The female part may then be put on and a thin piece of platinum cut out to slip down over the key next to the cap (Fig. 19) and burnished close to it. It is then waxed in position, removed with the female, and covered with coin gold. After which it may be replaced and the facings ground in. If a saddle is to be used, it is replaced, waxed to the saddle and soldered, and the facings ground in as before.

We now come to the description of the appliance which is practicable only in removable work. This is the saddle. By its use the possibilities of bridge-work are greatly increased. In the upper mouth, where there is sufficient anchorage in front, the bridge may be extended so as to carry one or two teeth, and in the lower even more. Where there are good abutments at either end, or near the ends, pieces of five, six, or more teeth may be made. In making a denture of this kind, the first thing to be done is to make the saddle. The impression may be trimmed to the size and shape wanted, and the model run leaving a ridge all around to where it should extend, or after the model has been made it may be built up with wax so as to make it the same. (Fig. 20.) An impression is then taken in moldine and a die and counter-die of fusible metal made.

The plate is then struck up of pure platinum of No. 28 or No. 30 gauge, with the edges slightly turned. (Fig. 21.) I will say at this point that the fusible metal, of which frequent mention is made, is prepared from a formula given by Dr. C. M. Richmond several years ago in the INTERNATIONAL DENTAL JOURNAL, and is composed of tin, twenty parts; lead, nineteen parts; cadmium, thirteen parts, and bismuth, forty-eight parts, making one hundred parts in all. These are melted in the order named. In my work I have found it indispensable and superior to any other fusible metal I have ever used. It melts at a low temperature, makes a clean die, is very hard, and, with a second die for finishing, almost any metal can be swaged.

The saddle being struck up is covered with coin gold to stiffen it and thicken the edges so that they will not injure the tissues. It is then fitted to the model again, and the surface of the coin ground smooth. If a telescope cap is to be used, they are both placed in position in the mouth, the saddle pressed firmly to place, and held there with an instrument while a plaster impression is taken, after which it is invested and soldered to the outer cap with twenty-carat solder. It is then placed in the mouth again, and an impression and bite taken in plaster. The inner cap is coated on the inside with a film of wax, and the model is made with the articulation as described earlier in this paper. The facings are now ground and placed so as to leave a little space between them and the saddle. Fig. 22 is backed with thin soft platinum which extends to the saddle and is waxed to it. The buccal side is then waxed and carved to represent the gum (Figs. 22 and 23), an impression taken, and a piece of pure gold twenty-four or twenty-six gauge swaged to fit around the teeth. The saddle is then heated slightly, which softens the wax and allows the facings to be removed without changing their position. The pure gold is soldered to the saddle, and the facings replaced. The cusps are then made, fitted, and the pieces are invested so as to leave the back exposed. (Fig. 24.) The wax is then carved to represent the lingual aspect of the teeth, an impression in moldine taken extending from the lower edge of the saddle to the upper surface of the cusps. (Fig. 24.) Make a die and counter-die and swage a back of No. 27 or 28 gauge coin gold and fit same accurately. (After swaging gold or platinum, it should always be dipped in nitric acid to remove any particles of the fusible metal which may be on the surface, as, if the piece is heated while any of it adheres to it, the gold would become slightly alloyed and so brittle as to be useless.) The wax is then removed, the piece heated up, and soldered with eighteen-carat solder, which is sufficiently fine, as none of it comes to the surface. Enough solder should be used to give all the strength required and to seal it perfectly and no more, as it only increases the weight. (Fig. 24.) The piece should be kept well heated and plenty of solder flowed over the parts which will come in contact with the back. The back is then dipped in flux and dropped in position.

The putting in of this piece is a very delicate operation, and requires the nicest manipulation, as in making the solder flow between the plates the back is very easily burned. However, if the operator is used to the blow-pipe, the piece kept very hot, and a large flame used, there is not much danger.

From this on there is very little trouble. The piece being sufficiently cooled, it is taken from the investment, cleaned in acid, the rough parts ground away, and the final touch before the polishing is given. It will be remembered that there has been a little space left between the facings and the saddle, and a piece of pure gold fitted around the necks of the teeth. (Fig. 22.) This space is filled with cement or plaster of Paris, and after it is thoroughly dried the pure gold is condensed around and against the facings with smooth pluggers and mallet, and the other spaces carefully filled with foil and burnished. The whole piece is then smoothed with fine corundum wheels, and the articulating most carefully done, as the success and comfort of the piece depends largely upon this. A poorly articulated piece, let it be never so well made and finished otherwise, will not prove satisfactory. The cusps are now carved, and the whole finished with pumice, abrada, and rouge. For reaching difficult places cork on an engine mandrel will be found useful. Also hard, thin, knife-edge disks for the lathe are indispensable. A bridge made in this manner comes as near being perfectly clean as one can be made, and if even ordinary care is given it will retain a clean polished surface, and there will never be the least disagreeable odor from it.

The thin, hard disks mentioned will be kept at the dental depots, or a number can be easily made in a very few minutes by the dentist himself. Put an ordinary felt wheel on the screw-chuck of the lathe, and with a thin, sharp knife cut while the lathe is running rapidly, starting near the edge and gradually increasing the thickness to one-eighth or three-sixteenths of an inch at the centre. Three or four can be made from an ordinary felt wheel. They are then dipped in white shellac varnish and dried on a piece of glass or board. When thoroughly dried, put on the chuck again, and while it is running apply a little heat, at the same time pressing the side with a small stick or a tooth-brush handle. In this way they can be made of any shape desired. When they are cool hold a coarse vulcanite file to the edge of the running disk and bring it to a sharp edge.

For abrada, a soft felt wheel is good, and the same will answer for rouge, having one very soft and thin for getting between the teeth. A soft cotton buff is the best for the final touch, with a brush wheel for the cusps.

The final step, and a highly important one, is the cementing of the piece. You are all familiar with it, so I will consider it briefly. The teeth, roots, and pieces to be cemented should be as dry as it is possible to make them. The cement is mixed to the consistency of thick cream, carried well up into the canals with a blunt instrument, the caps and bands filled, and the bridge forced quickly into position. Plenty of cement should be used, and the caps be perfectly tight, so as to force the cement to every part. If the edges of the band stand away from the neck at any place, it can be pressed in with a burnisher, and after the cement has hardened a little, the excess can be removed with an explorer. An opening should not be left in the cap to allow the surplus to escape, as is the custom with some, for the cement, instead of reaching every crevice, might find its way through this vent.

For this work I have found nothing equal to Weston's "cement for bridge-work." It sets very quickly, and the surplus can be removed from around the parts almost as soon as the piece has been put on. Those without experience would do well to mix two or three lots before trying to use it in the mouth. Otherwise they may get caught with a piece partly on which they can neither get to place nor remove.

It would do no harm to emphasize a few things which might prove disastrous. Do not expect too much of a tooth, and put a bridge of five or six teeth where there should be only three or four. Do not put a cap or band on until sure that the last bit of enamel has been removed. Do not use a pin one-quarter of an inch in length where it should be one-half inch. Do not in opening up the canal perforate the roots, and, lastly, do not put a piece in the mouth until it is properly articulated. By keeping in mind these points, a satisfactory bridge may be made which will last beyond the limit placed by some as the life of the work. A few minutes before penning these words, I read this following extract from a journal: "If a bridge lasts three or four years it has done all that could be expected of it, and the dentist has fulfilled his obligation to the patient." If it would last no longer than that it would certainly be a failure.

## Figures and Tables

**Fig. 1. Fig. 2. Fig. 3. Fig. 4. Fig. A. Fig. 5. Fig. 6. Fig. 7. Fig.8. Fig.9. Fig.10. Fig.11. Fig.12. Fig.13. Fig.14. Fig.15. Fig.16. Fig.17.&18. Fig.19. Fig.20. Fig.21. Fig.22. Fig.23. Fig.24. f1:**